# Coagulation Treatment of Wastewater: Kinetics and Natural Coagulant Evaluation

**DOI:** 10.3390/molecules26030698

**Published:** 2021-01-29

**Authors:** Nomthandazo Precious Sibiya, Sudesh Rathilal, Emmanuel Kweinor Tetteh

**Affiliations:** Green Engineering and Sustainability Research Group, Department of Chemical Engineering, Faculty of Engineering and the Built Environment, Durban University of Technology, Durban 4001, South Africa; nomtha.presh@gmail.com (N.P.S.); rathilals@dut.ac.za (S.R.)

**Keywords:** alum, eggshells, coagulation kinetics, ferromagnetite, magnetic coagulants, nanomaterials, wastewater treatment

## Abstract

In this study, three coagulants (ferromagnetite (F), alum (A), and eggshells (E)) and their hybrids (FA, FE, and FEA) were investigated as possible cost-effective coagulants for the treatment of industrial wastewater. Scanning electron microscopy (SEM) coupled with energy-dispersive X-ray (EDX) was used to characterize the morphological and elemental compositions of the coagulants. The effects of coagulant dosage (10–60 mg/L) and settling time were investigated for the removal of turbidity, color, and total suspended solids. A jar tester (JTL6) operating at conditions of 150 rpm for 2 min (rapid mixing) and 30 rpm for 15 min (slow mixing) was employed. Results from the characterized supernatant showed about 80% removal of the contaminants. The prospects of F were proven to be the most effective as compared to the binary (FA > FE) and the ternary hybridized (FEA) coagulants. At an optimum dosage and settling time of 20 mg/L and 30 min, respectively, the treatability performance of F was clearly proven to be viable for wastewater treatment.

## 1. Introduction

Water is a very important resource in agriculture, livestock production, forestry, fisheries, hydropower generation, industrial activities, and other innovation activities [1]. Most industrial effluents undergo some form of treatment and their characteristics depend on the manufacturing processes and types of raw materials used. Generally they contain levels of suspended solids that vary between 300 and 400 mg/L, with pH of 6.5–7.0, chemical oxygen demand (COD) of 2000–3000 mg/L, and total alkalinity of 50–100 mg/L. [2]. The production of huge amounts of heavily polluted wastewater has prompted research in order to develop, improve, and implement appropriate treatment techniques to eliminate pollutants [3]. Wastewater requires a post-treatment process to remove color and other organic pollutants prior to discharge into the nearest watercourse [4]. Well-established conventional methods for wastewater treatment include chemical precipitation, chemical oxidation or reduction, filtration, ion exchange, and the application of membrane technology [5,6].

Nevertheless, there are setbacks to these processes. These include incomplete metal removal, the need for expensive equipment and monitoring systems, high reagent and energy requirements, and/or the generation of toxic sludge or other waste products that require disposal [7]. According to Tetteh [8], the selection of a treatment technology depends on the recovered water quality, natural organic matter (NOM) size, wastewater chemistry, and discharge regulations. Normally, the coagulation process is utilized for the removal of suspended solids along with undesired or toxic substances [4] because it is simple, effective, and has low energy consumption [9,10]. It is known to be useful in protecting the environment and human health [11]. Aluminum and iron-based coagulants have been used in wastewater treatment. However, there are disadvantages connected with their use. These include the resulting end-of-life nanomaterials and increased wastewater color intensity as well as the generation of sludge and heavy metal residuals which are potentially toxic to the ecosystem [9,10,11,12,13,14]. The above-mentioned issues result in high wastewater treatment system costs as well as other technical disadvantages [15].

In the past decades, pre-treatment of industrial wastewater using coagulation and flocculation processes has become crucial to efficiently reduce the organic load prior to subsequent treatment processes [11,16]. Coagulation is an essential mechanism involving the addition of coagulants responsible for the destabilization and neutralization of suspended particles [17,18,19,20], which form large flocs or aggregates. Typically, negatively charged suspended particles agglomerate with positively charged coagulants due the adsorption of ions and ionization of surface groups [4]. These aggregates are removed by sedimentation, filtration, or flotation mechanisms [7,13]. Coagulation involves three different mechanisms: (1) charge neutralization (particle destabilization at low coagulant dosage), (2) sweep (the addition of coagulant at sufficiently high concentrations to cause anhydrous, amorphous precipitate enmeshing colloidal particles in these precipitates), and (3) bridge formation [14,21,22,23].

Coagulation processes do not comply with the stringent Environmental Protection Agency standards for regulating the quality of effluent plants [10,13] due to high energy consumption, high chemical costs, and the need for a second treatment for sludge to treat secondary pollutants [11,24]. Advancements in nanotechnology have contributed significantly to the development of the new techniques to resolve many health and environmental issues while using less energy [25,26,27]. Polymers have gained significant attention as an effective adsorbent for heavy metals due to their high affinity to bind with metal ions [11,28]. They are responsible for the production of larger, denser, stronger, and rapid-settling flocs [10]. In addition, they contain acrylamide monomers that are not harmful to humans and are unlikely to produce treated water with extreme pH and high biodegradability [12]. In addition, nanoparticles (Fe_3_O_4_) are increasingly considered to be significantly useful materials due to their specific properties, including their superparamagnetic, non-toxic nature and small size. [7,29].

Recently, magnetic technology has been gaining attention in the water and wastewater settings for remediation, removal of heavy metals, and separation of emerging contaminants [7,11,26]. The functionalization of magnetic iron oxide nanoparticles coupled with polymers such as eggshells, chitosan, rice starch, banana peels, moringa seeds, etc., is gaining attention in wastewater treatment settings [7,26]. This process is seen to be very attractive because of its high efficiency, capacity to reduce sludge volume, speedy sedimentation, and low cost [11,26,30]. There has been an upsurge in the use of biowaste materials like eggshell waste [31]. Eggshells are cost-effective, easily available as a biomaterial, and are widely used as bone substitutes, catalysts, and supports. They are efficient bio-templates due to their high catalytic activity, ease of handling, reusability, and benign character [32,33,34]. Ferromagnetite can be used as a coagulant in wastewater treatment due to its superparamagnetic properties that can influence the stability and adsorption capacity of the contaminants from wastewater [27,35]. According to Tetteh and Rathilal [7], the incorporation of ferromagnetite with alum improved the alum cluster content, inter-layer structures, surface area, and pore size, enhancing the wastewater treatability performance. Nevertheless, knowledge on the application of natural coagulants and ferromagnetite is limited.

Therefore, this study investigated the applicability of natural coagulants coupled with ferromagnetite in a magnetized coagulation system for wastewater treatment. This is foreseen to be economically viable, with additional benefits including wastewater reuse, reduced chemical costs, and minimized landfill and sludge-related problems [26,36]. This study investigated the performance of three coagulants (ferromagnetite (F), alum (A), and eggshells (E)) and their hybrids (FA, FE, and FEA) to identify a possible effective coagulant for the treatment of industrial wastewater. Morphological analysis of the coagulants was carried out. Furthermore, two (pseudo first-order and pseudo second-order) kinetic models were explored to validate the best fits of the experimental data.

## 2. Materials and Methods

### 2.1. Wastewater Samples

In this study, synthetic wastewater was simulated with the chemical compositions shown in Table 1. All the chemicals were of analytical grade and were evenly dissolved in 25 L of deionized water (ELGA PURELAB Option-Q water deionizer, UK) at room temperature [37]. Immediately after preparation, the initial sample concentrations were characterized in terms of turbidity (13.8 ± 0.94 NTU), color (47 ± 1.74 PtCo), and total suspended solids (TSS; 19 ± 0.24 mg/L). Turbidity was measured using a Hach 2100N turbidimeter, whereas color and TSS were analyzed by a spectrophotometer (HACH DR 3900, Germany). All the procedures used for analysis conformed to the standard methods of water and wastewater treatment [38].

### 2.2. Chemicals and Reagents

Aluminum sulfate (alum), ferric chloride hexahydrate, iron sulfate, and the rest of the chemicals in Table 1 were supplied by Sigma Aldrich. The eggshell powder was prepared according to the test method of Jagaba et al. [12]. Stock solutions of 0.1 M for each coagulant were prepared by dissolving 31.52 g and 25 g of alum (A) and eggshell (E), respectively, in a 1-L volumetric flask with deionized water. The ferromagnetite (F) was prepared via the co-precipitation method with a molar ratio of 1:2 for Fe (II):Fe (III) by respectively weighing 8.11 g and 15.15 g. This was then dissolved with 1 L of deionized water under slow mixing at 30 rpm for 1 h [12,13]. Subsequently, 2 mL of oleic acid was added as a surfactant followed by rapid mixing (150 rpm) at 80 °C for 2 h. The formed precipitate was allowed to cool and then filtered and washed with deionized water and ethanol. This was followed by 24 h of oven drying at 80 °C and calcination at 600 °C for 1 h. A scanning electronic microscope: Nova NanoSEM coupled with EDX and a Through-lens detector (TLD) was used to analyze the morphological structure of the powdered samples collected. This was operated at an acceleration voltage of 20 kV with a magnification in the range 1000k×.

### 2.3. Coagulation Tests

The coagulation experiment was done using a jar test (JTL6) apparatus coupled with six paddles. All experiments were performed using 500 mL of wastewater in beakers as per Maurya and Daverey [39]. Firstly, the dosage of 10–60 mg/L was investigated to obtain the desired dosages of A, E, and F, as well as the best combined dosage ratio as shown in Table 2. The effect of settling time (10–60 min) was also investigated at the specified optimum dosage attained. After addition of desired coagulants, the samples (S) were agitated with rapid mixing (150 rpm) for 2 min and slow mixing (30 rpm) for 15 min [7]. Thereafter, 15 min of motionless settling, the supernatants were collected with a syringe at 2 cm beneath the surface without disturbing the settled floc particles, and filtered (Whatman filter paper MN615 #90 nm). The treated sample was furthermore characterized for turbidity, color, and TSS, whereby the percentage removal was calculated using Equation (1).

(1) $$\%Removal\;efficiency\left(C_{n}\right)=\frac{C_{i}-C_{f}}{C_{i}}\times 100$$

where $C_{i}$ and $C_{f}$ are the initial and final values of each contaminant, respectively, and *C_n_* is the response parameter.

#### Agglomeration Kinetics

Coagulation is directed by Brownian motion of the suspended particles at an early stage [11,14]. Brownian motion becomes weak when colloidal particles destabilize and agglomerate to a diameter greater than 1 μm [35]. The kinetics of the coagulation process describe the rate of turbidity reduction, expressed by rate Equation (2) below [10,11,14,36]. Furthermore, the kinetics decide the rate of floc formation and assist in ending the critical time before the flocs destabilize. Kinetic study is very important because the rate of contaminant removal from the effluent depends on the kinetic parameters (*n* and *k*). The rate equation contains an independent variable (*t*), a dependent variable (*C*), and kinetic parameters.

(2) $$\frac{dC}{dt}=-kC^{n}$$

where *C* represents the concentration of particles, *t* is the coagulation time, *k* represents the *n*th order coagulation rate constant, and *n* is the order of the coagulation process. The rate constant is a product of the collision efficiency [11]. However, the particle concentration is indirectly proportional to time. The rate of contaminant removal can be directly proportional to the amount of contaminant concentration absorbed by the coagulant used [10]. The rate constant (Equation (3) is an outcome of the product of collision efficiency (*E*) and the Smoluchowski rate constant for a quick coagulation process $\left(K_{RC}\right)$ [40].

(3) $$k=E\times K_{RC}$$

where $K_{RC}$ is given by Equation (4):

(4) $$K_{RC}=\frac{4K_{B}T}{3\mu }$$

where μ-is the viscosity of the fluid.

The Brownian diffusion coefficient $\left(D_{B}\right)$ is given by Equation (5):

(5) $$D_{B}=\frac{K_{B}T}{\beta }$$

(6) β=2k

For the first order reaction (*n* = 1), Equation (2) becomes (7) when integrated:

(7) $$\ln\left(\frac{C}{C_{0}}\right)=k_{1}t$$

where $C_{0}$ and *C* represent the initial and final concentration (mg/L) of an effluent at *t* and $k_{1},$ which is the first order rate constant in 1/min. A plot of ln(CC0) versus *t* will yield a straight line passing through the origin with a slope of $k_{1}$ using Equation (7) [10,36,41]. Nevertheless, if the line does not cross the origin but goes through another y-intercept, it obeys the second-order coagulation process (n=2) where Equation (2) becomes Equation (8):

(8) $$\frac{dC}{dt}=-kC^{2}$$

Then, Equation (3) yields Equation (4) after integration:

(9) $$\frac{1}{C}=k_{2}t+\frac{1}{C_{0}}$$

where $k_{2}$ is the second-order rate constant in (Lmg·min).

## 3. Results and Discussions

This study investigated natural coagulants and their combination as an alternative to conventional coagulant (alum). The results obtained are presented in four sections, viz., the morphology of the coagulants (Section 3.1), the effect of coagulant dosage (Section 3.2), the effect of settling time (Section 3.3), and coagulation kinetics (Section 3.4).

### 3.1. Coagulants Morphological Results

**Figure 1:**
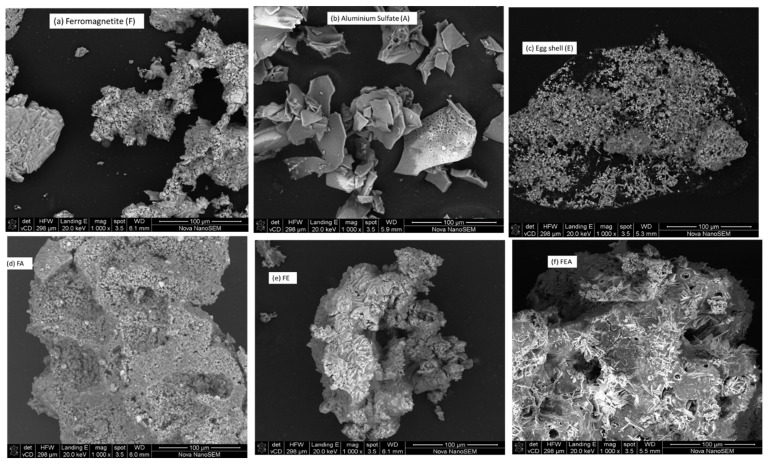
SEM images of: (**a**) Ferromagnetite (F), (**b**) alum (A), (**c**) eggshells (E), (**d**) FA, (**e**) FE, and (**f**) FEA.

**Figure 2:**
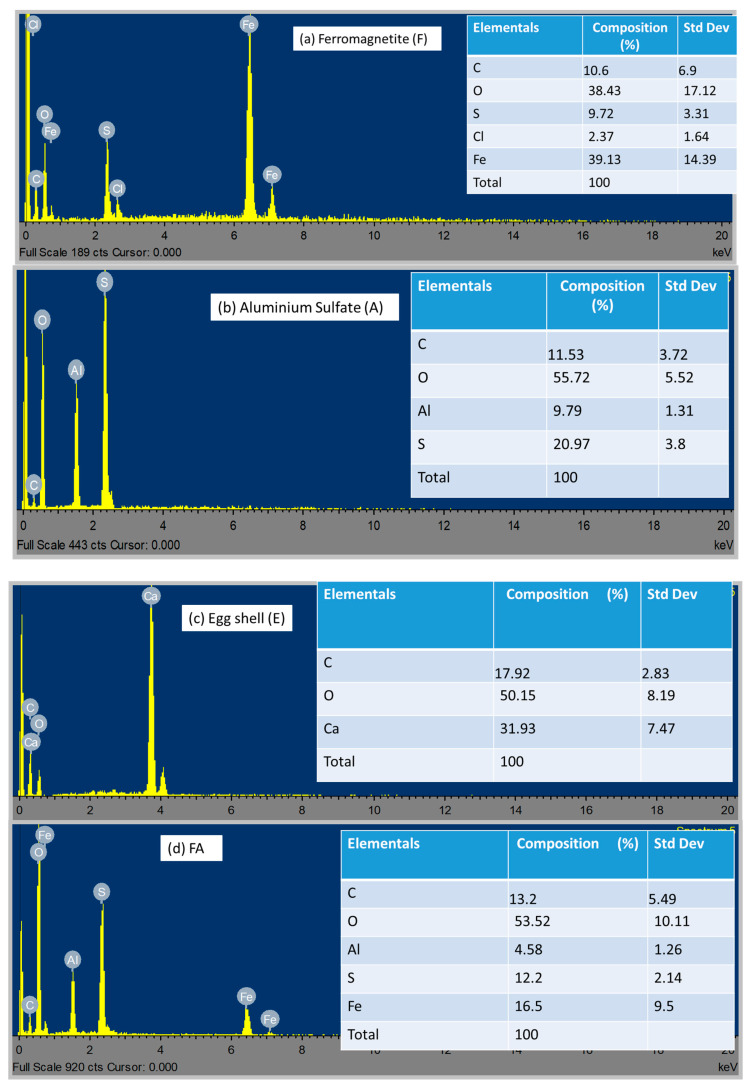

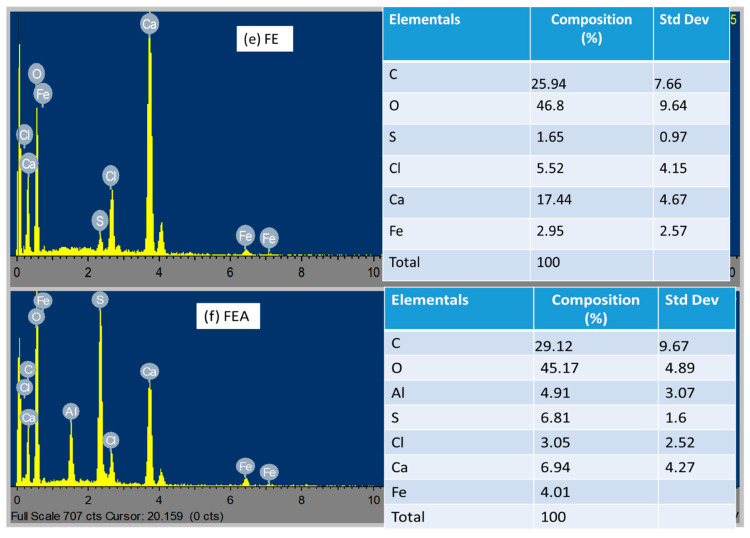
EDX image spectra of (**a**) ferromagnetite (F), (**b**) alum (A), (**c**) eggshells (E), (**d**) FA, (**e**) FE, and (**f**) FEA.

Surface morphological analysis of alum (A), eggshells (E), ferromagnetite (F), and their hybrids (FA, FE, and FEA) was carried out by scanning electron microcopy (SEM) coupled with energy-dispersive X-ray (EDX), to define their particle shapes and elemental distributions. Figure 1 shows the SEM images of the coagulant grains under the scale of 100 µm with magnification of 1000 kx and landing energy capacity of 20 keV. The hybridized images (Figure 1d–f) show rough surfaces with irregular shape and mesoporosity, with massive heterogeneity which enhanced the agglomeration of the large flocs [42,43]. Additional white droplet-shaped clusters with abundant pores were revealed on the extreme filmed surfaces (Figure 1a–c). In addition, hybridized images (Figure 1d–f) revealed a well-arranged surface with ideal knitted treads and microstructured strings, which can be attributed to the presence of the ferromagnetite as confirmed by the EDX (Figure 2). The additional whitish metaphors of numerous microspores on the surfaces (Figure 1d–f) could be due to the presence of calcium oxide and other impurities [44,45]. According to the working distances (WDs) of the SEM images (Figure 1d–f), the pore sizes of FEA (5.5 mm) were very small as compared to the other hybrid coagulants (FE (6.1 mm) > FA (6 mm)). Likewise, in their unmodified form (Figure 1a–c) the decreasing order of the pore size was as follows: F (6.1 mm) > A (5.9 mm) > E (5.3 mm). The EDX analyses of F, A, E, FE, FA, and FEA are shown in Figure 2a–f, with their corresponding elemental distributions. The F spectrum (Figure 2a) showed the composition Fe > O > C > S > Cl, whereas A (Figure 2b) contained the composition O > S > C > Al, and the composition O > Ca > C was found for E (Figure 2c). The hybridized macromolecules (FE, FA, and FEA) revealed high-affinity metallic ions with high selectivity, which enhanced the precipitation [46,47]. This affirmed that the morphological surfaces were well-bonded with the metallic ions for adsorption and agglomeration. Vepsäläinen [22] describes colloids as microscopic particles that range from 1 nm to 10 nm and disperse throughout the medium (liquid, gas, or solid). The total surface area of the dispersed colloids is large due to their small size [46]. The surface charge of colloids in the solution causes virtual stability in dispersions and destabilization when their sedimentation is slow [47,48,49]. The surface becomes charged through ionization of the functional groups (i.e., alcohol, carboxylic acid, and amine), ion adsorption, dissolution of ionic solids, and isomorphous substitution [48,49]. According to Duan and Gregory [23], colloidal stability and destabilization can be brought about by an increase in ionic strength with some reduction in the zeta potential, and a reduced thickness of the diffuse part of the electrical double layer.

### 3.2. Effect of Coagulant Dosage

**Figure 3:**
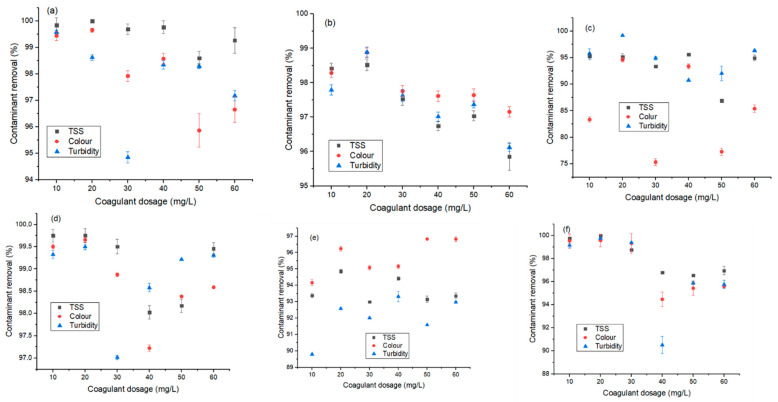
Effect of coagulant dosage (mg/L) on contaminant removal (TSS (black), color (red), and turbidity (blue)) using (**a**) A: alum, (**b**) E: eggshells, (**c**) F: ferromagnetite, (**d**) FE, (**e**) FA, and (**f**) FEA. TSS: total suspended solids.

This was a comparative study between three coagulants and their hybrids to obtain a possible cost-effective coagulant for the treatment of industrial wastewater. The jar test trials for each coagulant were carried out by varying the dosage in concentration (10–60 mg/L) in order to find the optimum dosage. The supernatants were decanted and their values for TSS, turbidity, and color were measured. Figure 3 presents the results obtained for the effect of coagulant dosage on the contaminant removal via the use of alum (A), eggshells (E), ferromagnetite (F), and their combined dosage (FA, FE and FEA). The agglomeration and destabilization of colloids (as in the treatment of the organic matter and hydrophobic organic matter) were seen to be better with the dosage of 10–20 mg/L. This affirms results of other studies that increasing the dosage of coagulant increases the treatability performance until agglomeration saturation is attained, whereby the performance starts to decline or stabilize [7,35]. Thus, an overdose of coagulant (>20 mg/L) as shown in Figure 3a–c,e–f reduced the treatability performance. This caused a remarkable reduction in contaminant removal (turbidity and color) due to the reversed net charge on the suspended solid in wastewater [7,40,41].

Furthermore, an overdose might have caused re-stabilization as a polymeric chain reaction could not occur, and the contaminant could have found empty sites for adsorption bridging [42,43], with an increased chance of sweeping [35]. In addition, overdosing or underdosing can affect the coagulation treatment process negatively, thereby increasing the cost of chemical usage [37,39]. In addition, the initial pH of the wastewater had an influence on the water–coagulant agglomeration chemistry. As reported by Sun et al. [35], at low pH (<7), the organic material in the water becomes negatively charged and is easily agglomerated. At a high pH (>7), there is a possibility of enhanced hydrophilicity as well as a reduction in the charge neutralization of the water molecules, coalescing ability, and treatability performance [35,37]. However, the intermolecular relationship between the turbidity and TSS removal was observed (Figure 3), as both decreased with a similar trend [35,44]. According to Mateus et al. [44], this phenomenon might be due to the alkaline affinity of the coagulant, as a similar reduction trend is observed for the removal of color. At an optimum dosage of 20 mg/L, the removal efficiency values for turbidity, color, and TSS were respectively recorded for each coagulant (A: 99.58%, 99.66%, and 100%; E: 98.89%, 98.89%, and 98.52%; F: 99.72%, 94.62%, and 95.19%; FA: 99.50%, 99.66%, and 99.75%; FE: 92.56%, 96.24%, and 94.86%; and FEA: 99.76%, 99.59% and 100%). While most of the coagulants showed better performance at a higher dosage (50 mg/L), due to the cost implication, a lower dosage (20 mg/L) was considered. Clearly, each coagulant showed unique performance with respect to contaminant removal. As such, FEA > FA > F > E > A were viable for turbidity and TSS removal, whereas FE maximized color removal. The efficiency of the combined coagulant was proven to be more effective due to the presence of the ferromagnetite, with unique magnetic properties and stability [7,45]. According to Tripathy and De [36], a suspension may be stable due to the influence of imbibed water molecules that provide a physical barrier that prevents the collision and destabilization of particulates. Electrostatic repulsion controls the stability of both hydrophobic and hydrophilic particles.

#### Comparative Study

**Figure 4:**
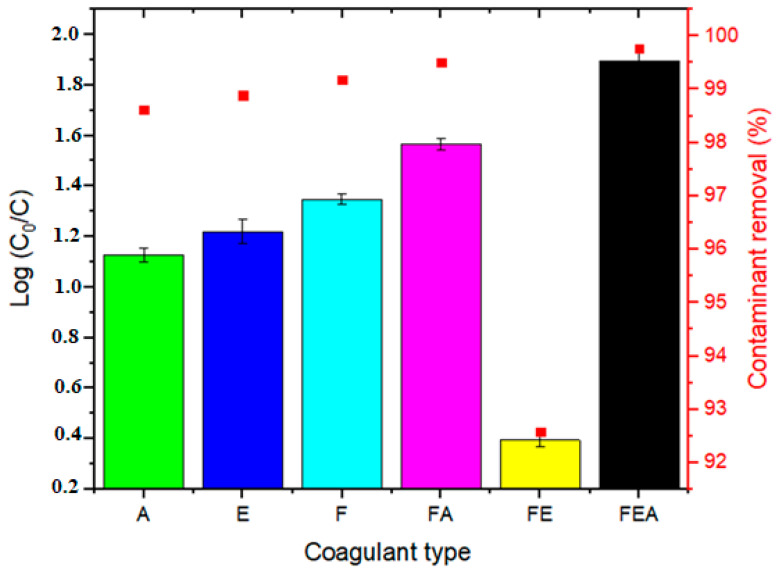
Performance of the coagulant at an optimum dosage of 20 mg/L for the removal of turbidity (%): FEA > FA > F > E > A > FE.

Figure 4 shows the comparative efficiency of the coagulants at the optimum dosage of 20 mg/L with regards to turbidity removal, where FEA showed the maximum removal efficiency. The order of coagulant performance was as follows: FEA (99.76%) > FA (99.51%) > F (99.17%) > E (98.89%) > A (98.62%) > FE (92.56%). As seen in Table 3, when comparing the performance of the coagulant to others in the literature, ferromagnetite showed great potential for future large-scale applications in the treatment of industrial wastewater [46].

### 3.3. Effect of Settling Time

**Figure 5:**
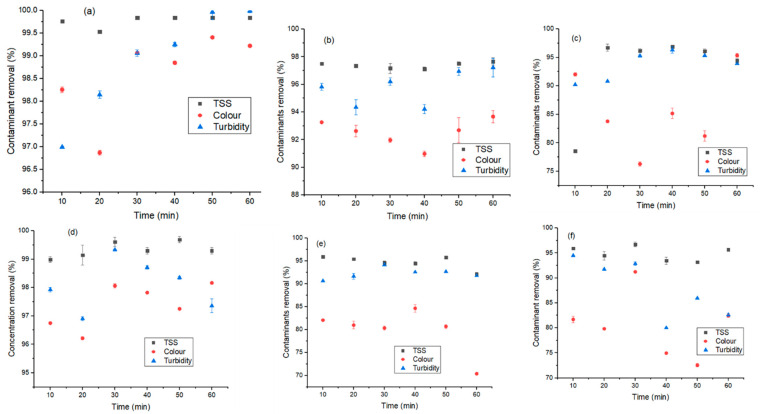
Effect of settling time (min) on contaminant removal (TSS (black), color (red), and turbidity (blue)) at 20 mg/L, using: (**a**) alum, (**b**) eggshells, (**c**) ferromagnetite, (**d**) FA, (**e**) FE, and (**f**) FEA.

**Figure 6:**
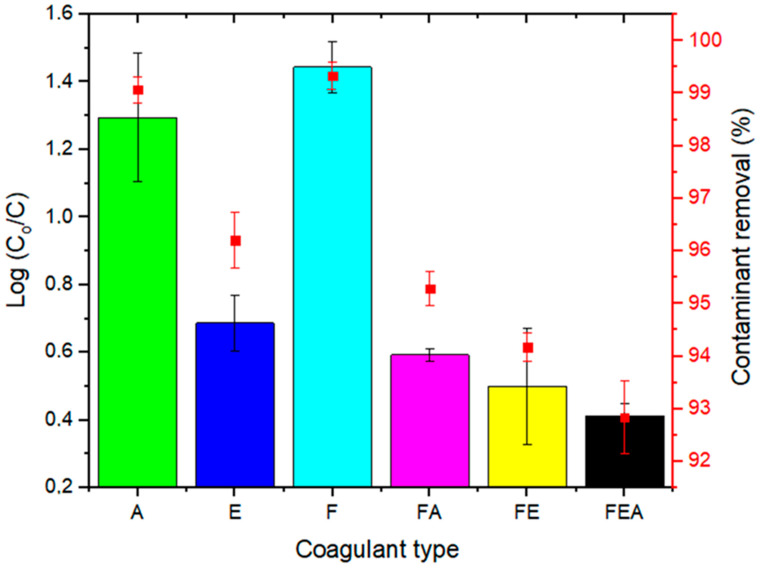
Performance of the coagulant at the optimum dosage (20 mg/L) and settling time (30 min) for the removal of turbidity (%): F > A > E > FE > FA > FEA.

To understand the coagulation–flocculation kinetics, the extent of aggregation was monitored. This was carried out by investigating the effect of sedimentation time (10–60 min) at a constant dosage of 20 mg/L for all three coagulants and their hybrids. Figure 5 reveals that a short duration (10–30 min) of the lag phase is desirable due to the energy and time savings [35,45]. Notwithstanding, a significant difference between the coagulants and the contaminants was observed with respect to an increase in the settling time. Figure 6 shows turbidity results for all coagulants at 30 min. Their order was as follows: F (99.34%) > A (99.07%) > E (96.21%) > FE (99.17%) > FA (92.85%) > FEA (91.74%). The growth of flocs and agglomeration was promoted, increasing the removal of color and TSS. However, with time, the floc size mostly decreased, with poor performance in turbidity removal. This was a result of the poor settling ability of the flocs due to the breakage of the colloids formed in suspension [50]. It was observed that all coagulants showed an exceptional TSS removal efficiency (>94%), followed by turbidity removal (>92%), and lastly color removal (>76%). The sharp reduction in turbidity with time (10–20 min) (for FEA, FA, and eggshell) reflects the fact that as the reaction progressed, the number of particles available for the coagulation was reduced. This may be due to the floc mechanism or combined bridging mechanism [45,50].

### 3.4. Coagulation Kinetics

**Figure 7:**
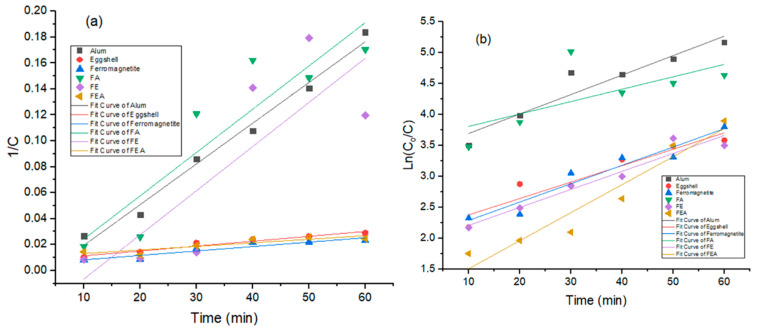
Kinetics of (**a**) second-order and (**b**) first-order models for the removal of turbidity.

The data obtained was fitted to kinetics Equations (7) and (9) to evaluate the performance for the treatment of the contaminants. The pollutant removal rate in a reaction-based system depends on numerous internal and external factors such as temperature, dosage, and the characteristics of wastewater itself [52]. When the reaction proceeds, pollutant particles attach themselves to both chemical and physical binding sites. The reaction lasts until complete saturation of free sites, with rapid settling. It was observed that the introduction of the ferromagnetite into the coagulants resulted in better agglomeration performance than a conventional coagulant (alum and eggshell) [11,51]. Thus, the data obtained was well fitted, with satisfactory significance for the applicability of the model for turbidity removal (Figure 7). It is evident (Table 4) that the coefficient of determination (R^2^) for the single coagulant dosage was favored by the second-order kinetic model (Figure 7a), whereas the first-order kinetic model (Figure 7b) showed a good fit for the combined coagulant dosage. In addition, the maximum removal of the contaminant corresponded with reasonable kinetic constants, as shown in Table 4. It could be suggested that the addition of F to other coagulants resulted in rapid floc agglomeration and greater floc size, which increased the coagulation efficiency and sedimentation. Thus, the extent of aggregation and flocculation dynamics were observed shortly after the addition of the coagulants, whereby the destabilized particles aggregated to form larger flocs [52]. Furthermore, the plateau of R-values revealed the degree of particle aggregation with respect to time taken for floc breakage to occur. The presence of floc breakage and the results obtained confirm that incorporation of the F increased the molecular weight of the coagulant components [50,52].

## 4. Conclusions

In this paper, the effect of coagulant dosage and settling time was investigated using three different coagulant types (alum (A), eggshell (E), and ferromagnetite (F)) and their hybrids (FA, FE, and FEA). The coagulant characteristics examined by SEM/EDX confirmed the presence of multivalent ions (Fe, Ca, Al) and their associated carbonates in the hybrids (FA > FE > FEA), which enhanced their adsorption and agglomeration capacity. The treatability performance showed over 80% removal of contaminants (turbidity, color, and TSS) at a settling time of 30 min and dosage of 20 mg/L. Evidently, ferromagnetite (F) and eggshells (E) as coagulants showed high potential for water and wastewater treatment as alternatives to conventional alum. The addition of the F and E in FA > FE > FEA coagulants resulted in rapid particle aggregation and larger floc size, resulting recommendable for enhanced flocculation and sedimentation in wastewater treatment processes. With regard to the kinetics results, magnetite-based coagulants (F, FA, FE, and FEA) exhibited greater floc formation and agglomeration. Therefore, the incorporation of F or E into coagulants and coagulation treatment was found to have potential applications in the wastewater setting.

## Figures and Tables

**Table 1:** Chemicals used and their quantity in the preparation of synthetic wastewater.

Chemicals Used	Quantity (g)
Peptone	4
Glucose	2.75
NaHCO_3_	27.5
Urea	0.75
Meat extract	6.25
MgSO_4_·7H_2_O	0.05
K_2_HPO_4_	0.7
CuCl_2_·2H_2_O	0.00125
NaCl	0.22
CaCl_2_·2H_2_O	0.1
Potassium silicate	1.5

**Table 2:** Combined dosage ratio of the coagulants.

Samples	FA (mg/L)		FE (mg/L)		FEA (mg/L)		
	F	A	F	E	F	A	E
S1	20	10	20	10	10	10	10
S2	20	20	20	20	20	10	10
S3	20	30	20	30	30	10	10
S4	20	40	20	40	40	10	10
S5	20	50	20	50	50	10	10
S6	20	60	20	60	60	10	10

* Ferromagnetite (F), alum (A), eggshell (E).

**Table 3:** Different coagulants used in wastewater treatment.

Coagulant Type	Dosage (mg/L)	Turbidity Removal (%)	Color Removal (%)	TSS Removal (%)	Reference
Eggshells (E)	20	98.89	98.88	98.52	This study
Alum (A)	20	99.58	99.66	100	This study
	50	70	75	-	[7]
Ferromagnetite (F)	20	99.17	94.62	95.19	This study
FEA	20	99.76	99.59	100	This study
FA	50	90	85	-	[7]
	20	99.51	99.66	99.75	This study
Alum–*Moringa oleifera*	43	97.70	-	-	[48]
Oyster mushroom bio- coagulant	600	84.10	-	90.69	[49]
Common bean	0.40	90.10	-	-	[50]
PAC and *Gossypium herbaceum* (GHC)	1600 and 2200	86.10 and 85.48	95 and 90.1	-	[51]
Walnut bio-coagulant	35	83	-	-	[52]

**Table 4:** Comparative kinetics study.

**First-order kinetics**						
Coagulant type	A	E	F	FE	FA	FEA
R^2^	0.9536	0.9523	0.9654	0.969	0.857	0.9659
Slope (k_1_)	0.0315	0.0265	0.296	0.201	0.0291	0.0453
y-intercept	3.38	3.11	1.99	3.61	1.92	1.05
SSE	0.0369	0.01172	0.02043	0.00945	0.01277	0.04292
**Second-order kinetic model**						
R^2^	0.993	0.979	0.978	0.9135	0.828	0.897
Slope(k_1_)	0.00314	0.000377	0.000341	0.00334	0.00341	0.0002756
y-intercept	−0.0119	0.00754	0.00487	−0.0091	−0.04057	0.01036
SSE	0.06269	0.0885	0.0374	0.041355	0.08397	0.02323

## Data Availability

Data is contained within the article.
